# COMMD2 Upregulation Mediated by an ncRNA Axis Correlates With an Unfavorable Prognosis and Tumor Immune Infiltration in Liver Hepatocellular Carcinoma

**DOI:** 10.3389/fonc.2022.853026

**Published:** 2022-04-29

**Authors:** Weidan Fang, Yu Gan, Ling Zhang, Jianping Xiong

**Affiliations:** Department of Oncology, The First Affiliated Hospital of Nanchang University, Nanchang, China

**Keywords:** COMMD2, CRNDE, LINC00511, SNHG17, HCG18, miR-29c-3p, tumor immune infiltration, liver hepatocellular carcinoma

## Abstract

Liver hepatocellular carcinoma (LIHC) seriously endangers the health and quality of life of individuals worldwide. Increasing evidence has underscored that the copper metabolism MURR1 domain (COMMD) family plays important roles in tumorigenesis. However, the specific role, biological function, mechanism and prognostic value of COMMD2 and its correlation with immune cell infiltration in LIHC remain unknown. In this study, we first determined the expression and prognostic potential of COMMD2 in human tumors using The Cancer Genome Atlas (TCGA) data and identified COMMD2 as a potential oncogene in LIHC. High COMMD2 expression was associated with pathological tumor stage and metastasis. Subsequently, noncoding RNAs (ncRNAs) upregulating COMMD2 expression were identified by performing expression, correlation, and survival analyses in combination. The CRNDE/LINC00511/SNHG17/HCG18-miR-29c-3p axis was identified as the most likely ncRNA-associated pathway upstream of COMMD2 in LIHC. Next, the expression profiles of COMMD2 and ncRNAs were validated in LIHC tissues and adjacent normal tissues. Furthermore, COMMD2 was significantly positively correlated with tumor immune cell infiltration, immune cell biomarkers, and immune checkpoint molecule expression. Importantly, COMMD2 potentially influenced prognosis by regulating immune cell infiltration in LIHC. Finally, COMMD2 was knocked down in LIHC cell lines using siRNAs for functional assays *in vitro*, resulting in suppressed cell proliferation and migration. In summary, our findings showed that the ncRNA-mediated upregulation of COMMD2 was associated with an unfavorable prognosis correlated with immune cell infiltration in LIHC.

## Introduction

Liver hepatocellular carcinoma (LIHC) is the most common type of primary cancer in the liver and third leading cause of cancer-related mortality worldwide (1). Although substantial improvements have been made in LIHC therapy, particularly in molecular targeted therapy and immunotherapy (2, 3), the 5-year survival rate of LIHC is dismal because of its high heterogeneity, complex genetics and clinical features (4, 5). Thus, molecular biomarkers urgently need to be identified to improve the prognosis and develop novel therapeutic strategies for LIHC.

The copper metabolism MURR1 domain (COMMD) protein family comprises 10 members (COMMD1–COMMD10), all of which share a structurally conserved C-terminal motif and are implicated in regulating many biological processes through protein-protein interactions (6). COMMD proteins are frequently dysregulated in various cancers and are associated with cancer progression and metastasis (7–11). For example, decreased COMMD1 expression increased tumor invasion (12) and suppressed the sensitivity of ovarian cancer cells to cisplatin (13). COMMD7 promotes cell proliferation, migration, and invasion by upregulating NF-κB signaling (14) or CXCL10 (15). COMMD9 promotes the progression of non-small cell lung cancer by increasing TFDP1/E2F1 activation (8). However, the expression, biological function, possible mechanism and prognostic relevance of COMMD2 and its correlation with immune cell infiltration in human cancers, including LIHC, remain unknown.

In this study, we first performed expression profiling and survival analysis of COMMD2 in various human cancers. Additionally, COMMD2 expression was correlated with cancer stage, tumor grade, lymph node metastasis and the TP53 mutation status in LIHC. Next, the mechanism underlying the regulation of COMMD2 by noncoding RNAs (ncRNAs), including long noncoding RNAs (lncRNAs) and microRNAs (miRNAs), through a competing endogenous RNA (ceRNA) network in LIHC was explored. Furthermore, we determined the correlations of COMMD2 with tumor-infiltrating immune cells, immune cell biomarkers, and immune checkpoint molecules in LIHC and analyzed the correlations between COMMD2 and infiltrating immune cells in the tumor microenvironment. Importantly, COMMD2 influenced the overall survival (OS) of LIHC patients through immune cell infiltration. Additionally, we performed a series of functional assays to further evaluate the effects of COMMD2 knockdown on LIHC cell proliferation and migration *in vitro*. Taken together, our findings suggest that the ncRNA-mediated upregulation of COMMD2 plays crucial roles in the development of LIHC by regulating immune cell infiltration.

## Materials And Methods

### Cell Culture and Transfection

The LIHC cell lines MHCC-97H and Huh-7 were purchased from the Cell Bank of the Chinese Academy of Sciences (Shanghai, China). MHCC-97H and Huh-7 cells were cultured in DMEM (HyClone, Logan, UT, USA) supplemented with 10% fetal bovine serum (FBS; Gibco, Grand Island, NY, USA) and 1% penicillin/streptomycin in a humidified incubator at 37°C with 5% CO_2_. COMMD2-targeting siRNA oligonucleotides were purchased from GenePharma (Shanghai, China). Cells were transfected with the indicated siRNAs using TurboFect transfection reagent (R0532; Thermo Scientific Scientific, Waltham, MA, United States). The siRNA sequences used in our study were as follows:

COMMD2-Homo-54: 5′-GGAAUUGUCCGAGGAGCAUTT-3′, COMMD2-Homo-222: 5′-GCAUGGUGUGGAAGGAUUATT-3′.

### Real-Time Quantitative PCR

Total RNA was extracted from cells and clinical samples using the acid guanidine method with TRIzol reagent (Thermo Fisher Scientific), chloroform and isopropanol according to the manufacturer’s instructions. cDNA was obtained by reverse transcription using the PrimeScript™ RT reagent kit (Takara, Dalian, China). Real-time quantitative PCR (RT–qPCR) was performed using TB Green™ Premix Ex Taq II (Takara, Dalian, China) on an CFX96 Real-Time PCR Detection System (Bio-Rad, USA) and following minimum standard MIQE guidelines (16). The RT–qPCR cycling conditions were as follows: 95°C for 30 s, 42 cycles at 95°C for 5 s, and 60°C for 30 s. The melt curve stage was set as follows: 95°C for 15 s, 60°C for 60 s, and 95°C for 15 s. The primer sequences are shown in **Table 1**. The miRNA, mRNA and lncRNA levels were normalized to those of U6 or GAPDH. The relative expression level of mRNA from cells was calculated using the 2^−ΔΔCT^ method and the miRNA, mRNA and lncRNA levels from clinical samples was assessed using the 2^−ΔCt^ method.

**Table 1:** Primers used in the study.

Primer	Forward (5’-3’)	Reverse (5’-3’)
COMMD2	TGAATTGGCACCAAGCCTTC	TGGGTCTGTCTGCAGAACTT
CRNDE	ATATTCAGCCGTTGGTCTTTGA	TCTGCGTGACAACTGAGGATTT
LINC00511	AGGGGCGACTACTGTTACCT	CGTCCAAACAGGCTGGATCT
SNHG17	TTTTCCCACGCTGTCTGTCA	CAGTTTCCCCCGATGGTGAG
HCG18	ATCCTGCCAATAGATGCTGCTCAC	AGCCACCTTGGTCTCCAGTCTC
miR-29c-3p	AACACGTGTAGCACCATTTGAA	CAGTGCAGGGTCCGAGGT
GAPDH	ATGGGGAAGGTGAAGGTCG	GGGGTCATTGATGGCAACAATA
U6	CGCTTCGGCAGCACATATAC	TTCACGAATTTGCGTGTCATC

### Tissue Samples and Ethical Statement

Fresh LIHC specimens and adjacent normal tissue were obtained from 10 LIHC patients who were undergoing surgery at the First Affiliated Hospital of Nanchang University (Nanchang, China). The patients were treatment-naïve before surgery. This study was approved by the Ethics Committee of the First Affiliated Hospital of Nanchang University.

### CCK8 Viability and Clone Formation Assays

Cells were seeded in 96-well plates at a density of 2 × 10^3^ cells per well, after which 100 μl of FBS-free medium containing 10% CCK8 was added to each well and incubated for 2 h at 37°C. Next, the OD values at 450 nm were detected using a microplate reader (Thermo Scientific) for 5 days. For the clone formation assay, cells were seeded in 6-well plates at a density of 2 × 10^3^ cells per well and cultured for 14 days. Next, the clones were fixed with methanol, stained with 1% crystal violet and counted.

### Wound Healing Assay

Cells were seeded in 6-well plates and cultured in serum-free DMEM to 100% density, after which wounds were created by scratching with 10 μl pipette tips. Images were acquired at 0 and 24 h, and the wound areas were quantified using ImageJ software.

### Cell Migration Assay

Cells were seeded on the upper transwell chamber at a density of 3 × 10^4^ cells in 200 μl of serum-free culture medium, and 600 μl of medium containing 20% FBS was added to the lower chamber. After 48 h, the cells that migrated through the membranes were fixed with methanol, stained with 1% crystal violet and counted under a light microscope.

### Data Acquisition and Processing

We downloaded LIHC-related mRNA-seq expression profiles from The Cancer Genome Atlas (TCGA) database (https://portal.gdc.Cancer.gov/). Additionally, the expression profiling data of the arrays GSE55092 and GSE107170 (GPL570 sequencing platform) from the GEO database (http://www.ncbi.nlm.nih.gov/geo/) were downloaded as validation datasets.

### Immune Infiltration Analysis

TIMER (https://cistrome.shinyapps.io/timer/) is an online database used to comprehensively analyze tumor-infiltrating immune cells in various cancer types (17). The expression levels of COMMD2 in multiple cancers and their correlation with immune cell infiltration or immune checkpoint molecules in LIHC were analyzed using TIMER. CIBERSORT is a deconvolution algorithm based on gene expression that assesses the relative variations in immune cell infiltration (18). The immune infiltration levels of 22 immune cell types in patients in the TCGA-LIHC cohort, GSE55092 and GSE107170 were analyzed using the CIBERSORT algorithm. A p value <0.05 was considered to be statistically significant.

### UALCAN Analysis

UALCAN (http://ualcan.path.uab.edu) is a comprehensive and interactive online tool that includes 31 cancer types from the TCGA database (19). In this study, COMMD2 mRNA expression in various cancer types and its association with survival prognosis were analyzed. Furthermore, UALCAN was used to analyze the associations of COMMD2 with clinicopathologic parameters, such as patient sex, patient age, cancer stage, tumor grade, nodal metastasis status and the TP53 mutation status in LIHC. A p value <0.05 was considered to be statistically significant.

### GEPIA Database Analysis

GEPIA (http://gepia.cancer-pku.cn/) is an interactive analysis online tool for cancer and normal gene expression profiling using TCGA and Genotype-Tissue Expression (GTEx) data (20). GEPIA was used to determine and assess the expression and prognostic values of candidate lncRNAs in LIHC. Additionally, the correlations between COMMD2 and immune checkpoint molecules in LIHC were evaluated. A p value <0.05 was considered to be statistically significant.

### Candidate miRNA Prediction

miRNAs binding upstream of COMMD2 were predicted using the TarBase (http://www.microrna.gr/tarbase), miRTarBase (http://mirtarbase.mbc.nctu.edu.tw//), miRWalk (http://mirwalk.uni-hd.de/) and starBase (http://starbase.sysu.edu.cn/) databases. Only the predicted miRNAs that commonly appeared in more than two of the abovementioned databases were considered candidate miRNAs of COMMD2.

### StarBase Database Analysis

starBase is an online database for exploring miRNA, lncRNA and RNA interaction networks (21). starBase was used to conduct the following correlation analyses in LIHC: miRNA-COMMD2, lncRNA-miR-29c-3p and lncRNA-COMMD2. The expression of candidate miRNAs in LIHC was also analyzed. Additionally, starBase was used to predict candidate lncRNAs that could potentially bind to miR-29c-3p. A p value <0.05 was considered statistically significant.

### Kaplan-Meier Plotter Analysis

Kaplan-Meier plotter (http://kmplot.com/analysis/), an online database containing data on the relationships between gene or miRNA expression and clinical outcomes in more than 20 cancer types, was used to assess associations of miR-29c-3p with the survival of LIHC patients and the associations of COMMD2 with patient survival in various cancer types. OS and progression-free survival (PFS) with hazard ratios (HRs) with 95% confidence intervals (95% CIs) and log-rank p value were evaluated (22).

### Statistical Analysis

The statistical analyses in this study were automatically performed using the above online databases. Paired Student’s t test was used to evaluate the differences in ncRNA and COMMD2 expression between the cancer and control groups. Student’s t test was used for comparisons. P<0.05 was considered statistically significant. * P<0.05 and ** P<0.01.

## Results

### Expression Levels of COMMD2 in Multiple Cancers

**Figure 1:**
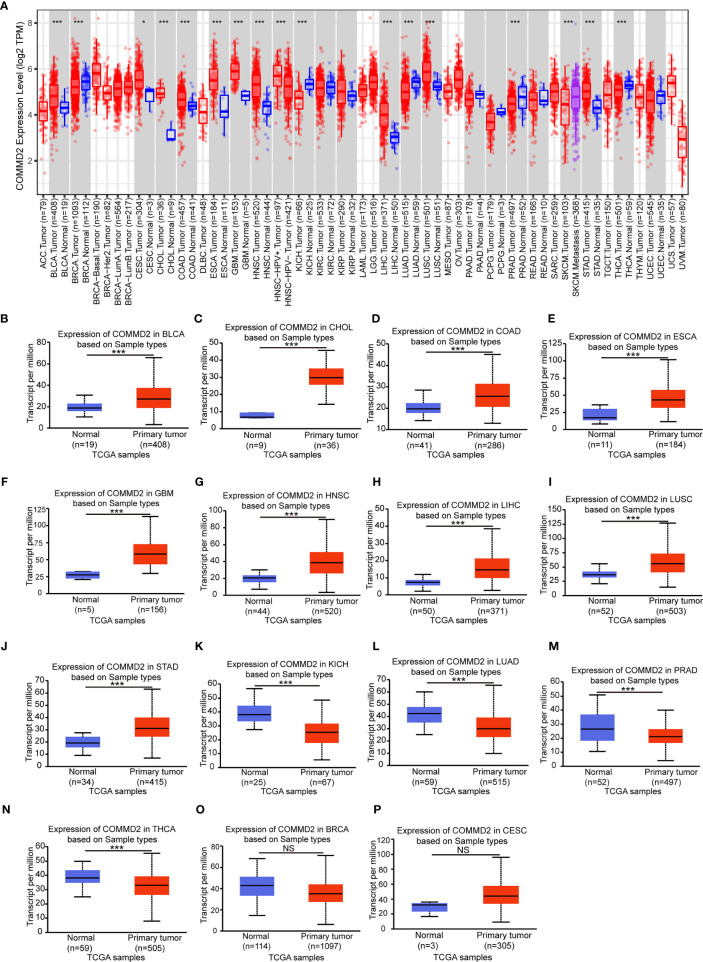
Expression levels of COMMD2 in different cancer types. **(A)** COMMD2 expression in different types of cancer was determined using the TIMER database. **(B–M)** COMMD2 expression in BLCA **(B)**, CHOL **(C)**, COAD **(D)**, ESCA **(E)**, GBM **(F)** HNSC **(G)**, LIHC **(H)**, LUSC **(I)**, STAD **(J)**, KICH **(K)**, LUAD **(L)**, PRAD **(M)**, THCA **(N),** BRCA (**O**) and CESC **(P)** tissues compared with corresponding normal tissues as determined using the UALCAN database. *p value < 0.05, **p value < 0.01, ***p value < 0.001. NS, Not Significant.

To investigate the possible roles of COMMD2 in tumorigenesis, its expression levels in tumor and normal tissue samples of multiple cancer types were analyzed using the TIMER database. COMMD2 was expressed at significantly higher levels in the tumor tissues of 10 various cancer types, including bladder cancer (BLCA), cervical squamous cell carcinoma (CESC), cholangiocarcinoma (CHOL), colorectal adenocarcinoma (COAD), esophageal cancer (ESCA), glioblastoma multiforme (GBM), head and neck squamous cell carcinoma (HNSC), LIHC, lung squamous cell carcinoma (LUSC) and stomach adenocarcinoma (STAD), than in the corresponding normal tissues. However, in breast cancer (BRCA), kidney chromophobe (KICH), lung adenocarcinoma (LUAD), prostate adenocarcinoma (PRAD) and thyroid cancer (THCA) tissues, COMMD2 expression was markedly lower than that in the corresponding normal samples (**Figure 1A**). To further evaluate COMMD2 expression in human cancers, the UALCAN database was evaluated, revealing that the COMMD2 expression levels in BLCA, CHOL, COAD, ESCA, GBM, HNSC, LIHC, LUSC and STAD tissues were significantly higher than those in the corresponding normal tissues (**Figures 1B–J**). However, compared with its levels in corresponding normal tissues, the COMMD2 expression levels in KICH, LUAD, PRAD and THCA tissues were obviously lower (**Figures 1K–N**). COMMD2 expression levels in BRCA and CESC tissues has no significantly difference than its levels in corresponding normal tissues (**Figures 1O, P**). Overall, COMMD2 expression was upregulated in BLCA, CHOL, COAD, ESCA, GBM, HNSC, LIHC, LUSC and STAD tissues and downregulated in KICH, LUAD, PRAD and THCA tissues, indicating that COMMD2 may crucially regulate carcinogenesis in these 13 types of cancer.

### Prognostic Value of COMMD2 in Various Human Cancers

**Figure 2:**
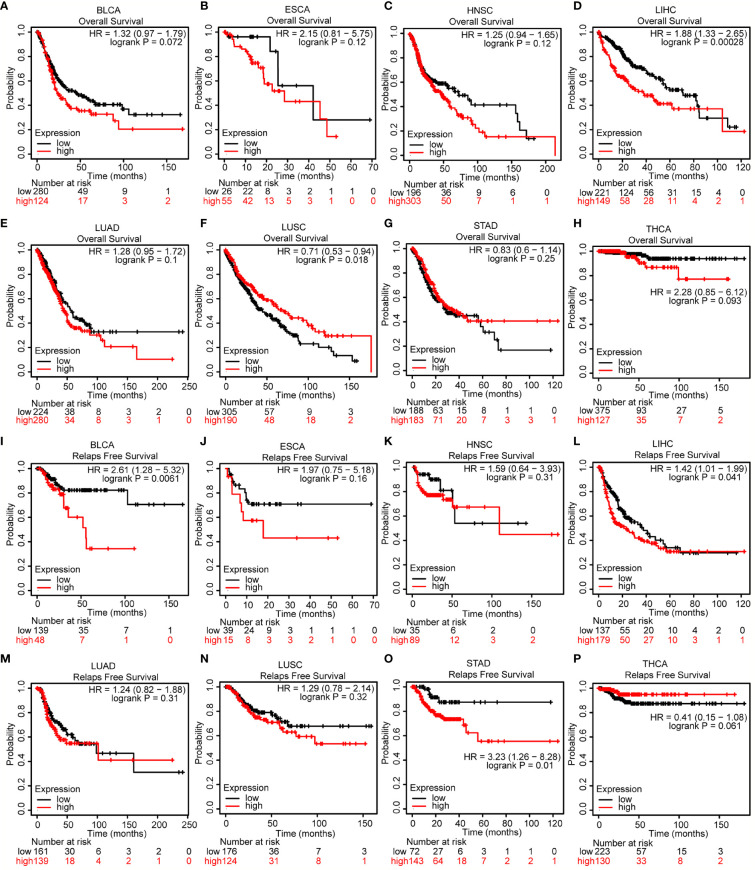
Prognostic value of COMMD2 in various human cancers. **(A–H)** Overall survival (OS) analysis of COMMD2 in BLCA **(A)**, ESCA **(B)**, HNSC **(C)**, LIHC **(D)**, LUAD **(E)**, LUSC **(F)**, STAD **(G)** and THCA **(H)**. **(I–P)** Relapse-free survival (RFS) analysis of COMMD2 in BLCA **(I)**, ESCA **(J)**, HNSC **(K)**, LIHC **(L)**, LUAD **(M)**, LUSC **(N)**, STAD **(O)** and THCA **(P)** .

The association between COMMD2 expression and prognosis was analyzed for various candidate types of cancer using the UALCAN database (**Supplementary Figure 1**). Notably, higher COMMD2 expression was significantly associated with a worse prognosis in LIHC (p= 0.0051, **Supplementary Figure 1F**), while higher expression of COMMD2 in CHOL indicated a better prognosis (p= 0.032, **Supplementary Figure 1B**). To further examine the prognostic potential of COMMD2 in different cancers, the Kaplan-Meier plotter database was used. Regarding OS, COMMD2 upregulation was associated with an unfavorable prognosis in LIHC (p= 0.00028; **Figure 2D**), but a higher level of COMMD2 was significantly associated with a positive prognosis in LUSC (p= 0.018; **Figure 2F**). Increased expression of COMMD2 was significantly correlated with short relapse-free survival (RFS) in BLCA, LIHC and STAD (**Figures 2I, L, O**). In other cancer types (**Figures 2A–C, E, G–H, J–K, M–N, P**), no significant correlations were found between COMMD2 and patient prognosis. In addition, the protein expression of COMMD2 was explored with the Human Protein Atlas (HPA) database. Similarly, COMMD2 was overexpressed in LIHC tissues compared with that in normal hepatic tissue. Higher COMMD2 expression was markedly correlated with a worse OS in LIHC patients (**Supplementary Figures 2A, B****)**. By combining the prognostic values from various databases, we concluded that COMMD2 might be an unfavorable prognostic biomarker for LIHC.

### Association of COMMD2 Expression With the Clinicopathological Features of LIHC Patients

**Figure 3:**
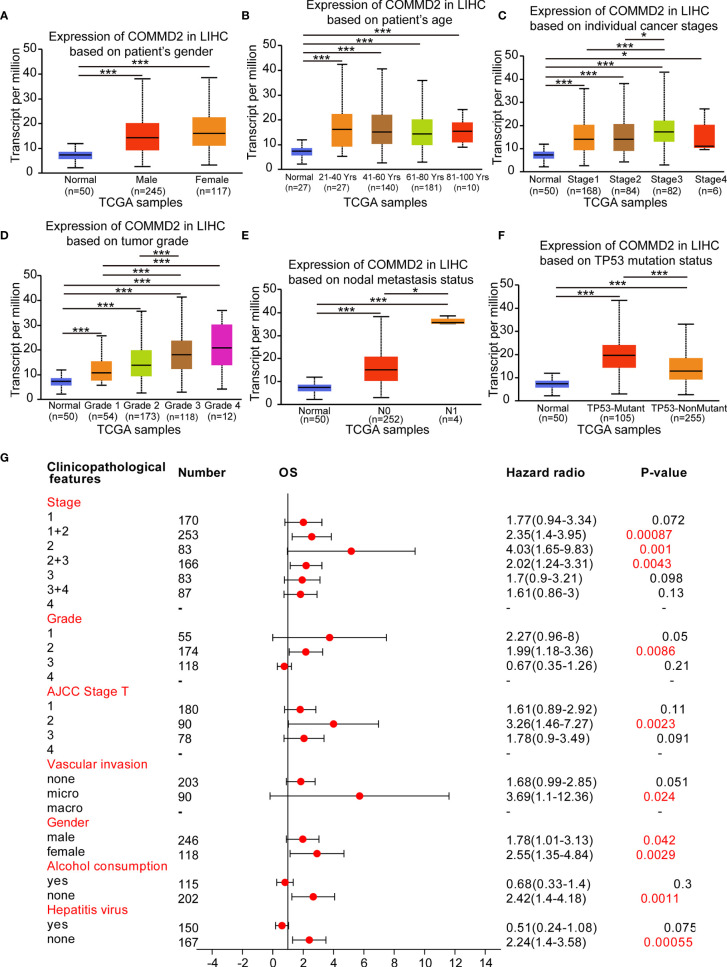
Association of COMMD2 expression with the clinicopathological features of LIHC patients. **(A–F)** Boxplot showing COMMD2 expression in normal individuals and LIHC patients based on clinicopathological features using the UALCAN database. Patient sex **(A)**, patient age **(B)**, cancer stage **(C)**, tumor grade **(D)**, nodal metastasis status **(E)** and TP53 mutation status parameters were analyzed **(F)**. **(G)** Forest plot showing the association between COMMD2 expression and clinicopathological parameters of LIHC patients. *p < 0.05; ***p < 0.001.

The relationships between COMMD2 expression and the clinicopathological parameters of LIHC patients, including patient sex, patient age, cancer stage, tumor grade, nodal metastasis status and the TP53 mutation status, were analyzed using UALCAN database. Regarding sex, COMMD2 expression was significantly upregulated in the LIHC tissues of both male and female patients compared with that in the normal tissues (**Figure 3A**). The COMMD2 level was significantly related to the age of the patient (**Figure 3B**) and remarkably correlated with cancer stage. Compared with that in normal tissues, COMMD2 expression was significantly higher in stage 1, stage 2, stage 3 and stage 4 cancers (**Figure 3C**). Concerning tumor grade, upregulation of COMMD2 expression was observed in grade 1, grade 2, grade 3, and grade 4 tumors, and COMMD2 expression increased as the pathological grade increased (**Figure 3D**). Moreover, COMMD2 expression was significantly related to the nodal metastasis status (**Figure 3E**). Furthermore, COMMD2 was expressed at a significantly higher level in the TP53 mutant than in the TP53 wild-type (**Figure 3F**). Additionally, to better understand the prognostic value of COMMD2 expression in LIHC, we explored the association between COMMD2 expression and clinical characteristics using the Kaplan-Meier database. High COMMD2 expression was significantly correlated with a poor OS in male and female patients with LIHC. Regarding the different tumor stages, upregulation of COMMD2 expression was associated with the a poor OS of patients with LIHC classified as stage 1 + 2, stage 2, and stage 2 + 3. A significant correlation between COMMD2 expression and a poor OS was observed in patients with American Joint Committee on Cancer (AJCC) stage T-2 and grade 2 LIHC. Additionally, high COMMD2 expression was significantly associated with an unfavorable OS for LIHC patients with microvascular invasion, patients who did not consume alcohol and patients without hepatitis. These results imply that COMMD2 expression possesses prognostic value in LIHC (**Figure 3G**).

### Prediction and Analysis of miRNAs Upstream of COMMD2

**Figure 4:**
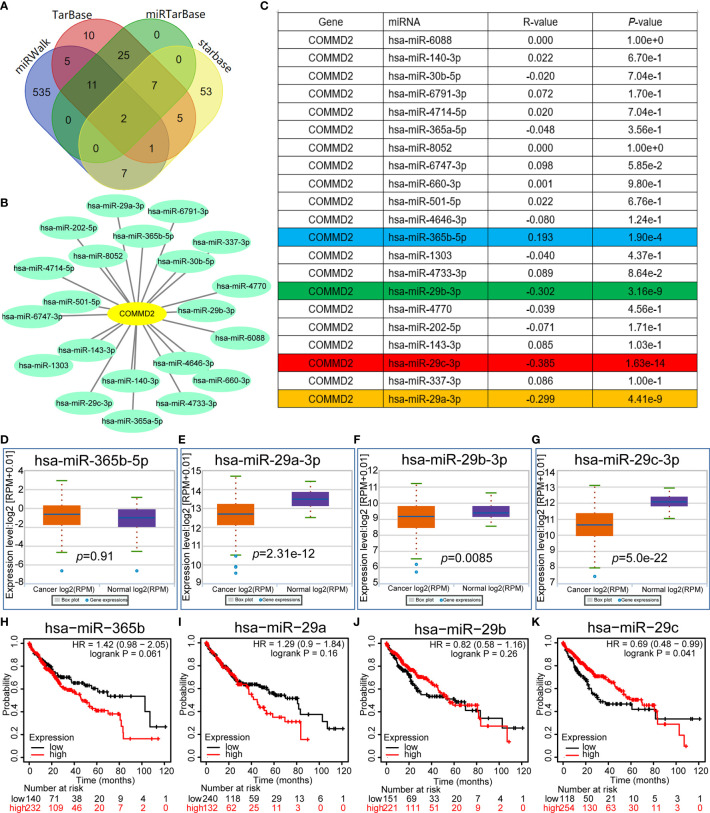
Prediction and analysis of miRNAs upstream of COMMD2. **(A)** miRNAs predicted to be upstream of COMMD2 using the miRWalk, TarBase, miRTarBase and starBase databases. **(B)** miRNA-COMMD2 regulatory network established using Cytoscape software. **(C)** Correlations between the predicted miRNAs and COMMD2 in LIHC as determined by the starBase database. **(D–G)** Expression of miR-365b-5p **(D)**, miR-29a-3p **(E)**, miR-29b-3p **(F)** and miR-29c-3p **(G)** in LIHC and normal tissue samples as determined by the starBase database. **(H–K)** Prognostic values of miR-365b-5p **(H)**, miR-29a-3p **(I)**, miR-29b-3p **(J)** and miR-29c-3p **(K)** in LIHC as assessed by Kaplan-Meier plotter.

Increasing evidence has shown that ncRNAs play key roles in the development of cancers by regulating gene expression. To determine whether COMMD2 is modulated by ncRNAs, the miRNAs binding upstream of COMMD2 were predicted by several target gene prediction programs, and 21 candidate miRNAs were ultimately identified (**Figure 4A**). The LIHC-associated miRNA-COMMD2 regulatory network was established using Cytoscape software (**Figure 4B**). According to the mechanism by which miRNAs generally negatively regulate downstream target genes, miRNAs and COMMD2 should be negatively correlated. Therefore, correlation analysis was conducted. COMMD2 was significantly negatively correlated with miR-29a-3p, miR-29b-3p and miR-29c-3p and positively correlated with miR-365b-5p in LIHC (**Figure 4C**). No significant relationships were observed between the expression of COMMD2 and other 17 predicted miRNAs. Finally, the expression and prognostic values of miR-29a-3p, miR-29b-3p, miR-29c-3p and miR-365b-5p in LIHC were determined. As presented in **Figures 4D–G**, miR-29a-3p, miR-29b-3p and miR-29c-3p were markedly downregulated in LIHC, but no significant upregulation of miR-365b-5p in LIHC was observed. Furthermore, only the high expression of miR-29c-3p was associated with a positive prognosis for LIHC patients (**Figures 4H** **–K**). Thus, miR-29c-3p might be the most promising miRNA that regulates COMMD2 in LIHC.

### Prediction and Analysis of LncRNAs Upstream of miR-29c-3p

**Figure 5:**
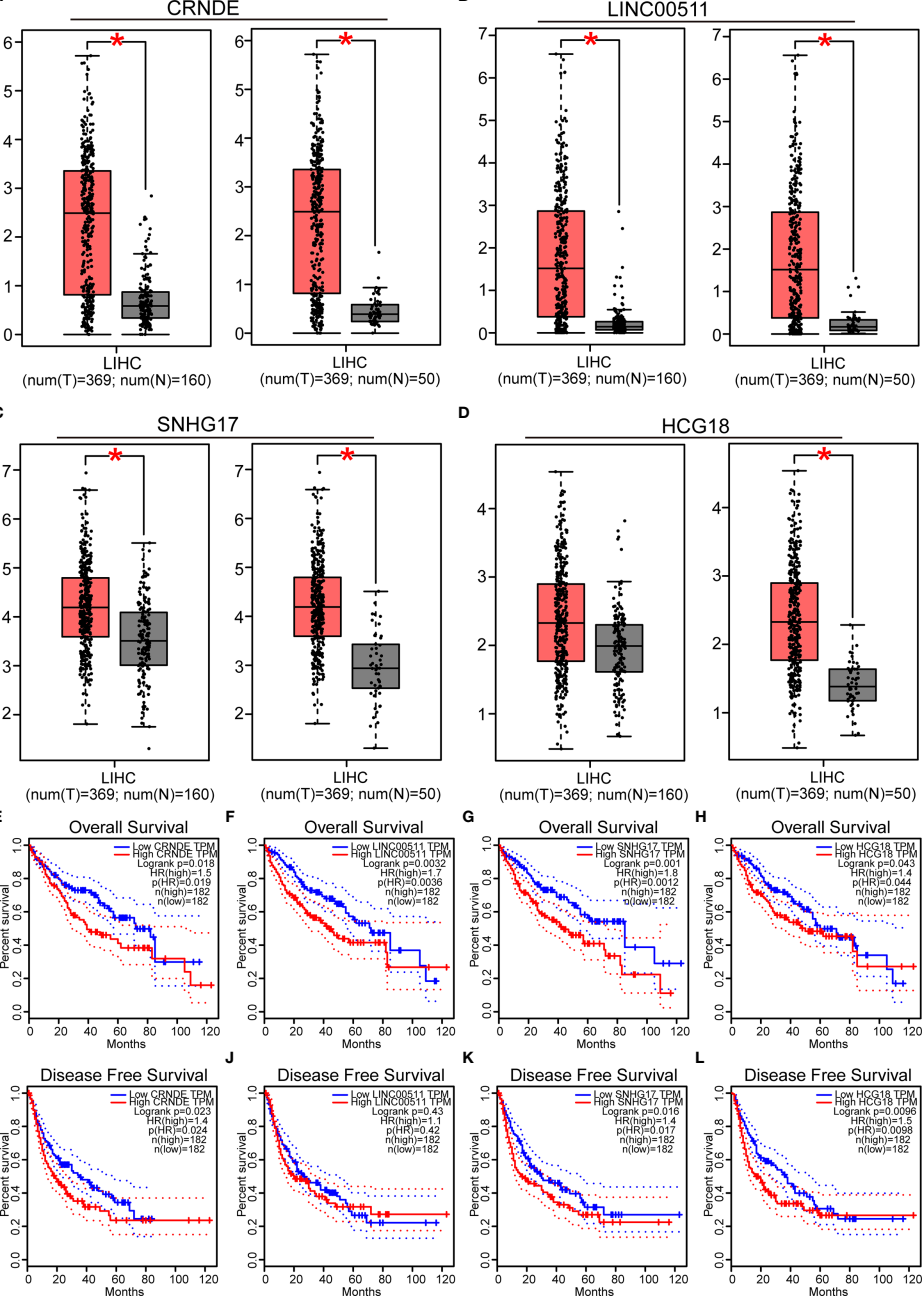
Prediction and analysis of lncRNAs upstream of miR-29c-3p. **(A–D)** Expression of CRNDE **(A)**, LINC00511 **(B)**, SNHG17 **(C)** and HCG18 **(D)** in LIHC data compared with that in “TCGA normal” or “TCGA and GTEx normal” data. **(E–L)** OS analysis of CRNDE **(E)**, LINC00511 **(F)**, SNHG17 **(G)** and HCG18 **(H)** in LIHC. RFS analysis of CRNDE **(I)**, LINC00511 **(J)**, SNHG17 **(K)** and HCG18 **(L)** in LIHC. ∗p value < 0.05.

**Figure 6:**
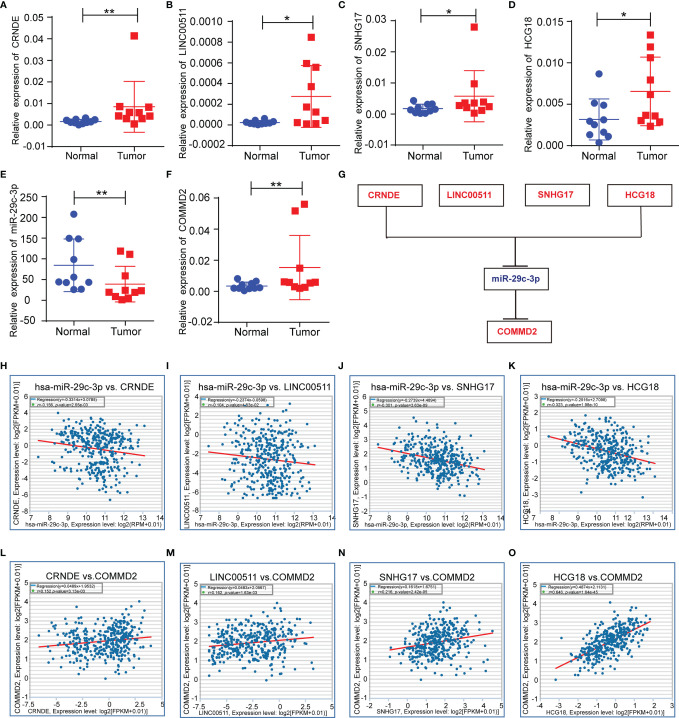
Correlations of lncRNAs with miR-29c-3p and COMMD2 in LIHC. **(A–F)** Expression of crucial ncRNAs and COMMD2 in fresh LIHC specimens and adjacent normal tissue as determined by RT-qPCR. **(G)** Schematic model of the ceRNA network. Red indicates upregulation and blue indicates downregulation. **(H–K)** Correlations of CRNDE **(H)**, LINC00511 **(I)**, SNHG17 **(J)** and HCG18 **(K)** with miR-29c-3p in LIHC. **(L–O)** Correlations of CRNDE **(L)**, LINC00511 **(M)**, SNHG17 **(N)** and HCG18 **(O)** with COMMD2 in LIHC. *p < 0.05; **p < 0.01.

Next, the lncRNAs upstream of miR-29c-3p were predicted using the starBase database. Fifty-four possible lncRNAs were predicted, and an lncRNA-miR-29c-3p regulatory network was constructed using Cytoscape software (**Supplementary Figure 3**). Next, the expression of these lncRNAs in LIHC was determined using GEPIA. Among the 54 predicted lncRNAs, only CRNDE, LINC00511, SNHG17, and HCG18 were expressed at significantly higher levels in LIHC tissues than in normal tissues (**Figures 5A–D**). Subsequently, the associations between the four lncRNAs and prognosis of LIHC patients were evaluated. High expression of CRNDE, SNHG17, or HCG18 was significantly associated with both unfavorable OS and disease-free survival (DFS) in LIHC patients (**Figures 5E–L**). In addition, increased expression of LINC00511 indicated a poor OS. Additionally, to further confirm the expression of ncRNAs and COMMD2 in LIHC, RT-qPCR was performed in 10 pairs of fresh LIHC specimens and adjacent normal tissue. In accordance with our previous analytic data, miR-29c-3p was significantly decreased in LIHC tissue compared with those in adjacent normal tissue (**Figure 6E**). In contrast, the expression levels of COMMD2, CRNDE, LINC00511, SNHG17 and HCG18 were significantly increased in LIHC tissue compared with adjacent normal tissue (**Figures 6A–D, F****)**. Based on the known interactions of these ncRNAs in ceRNA networks, lncRNAs potentially promote mRNA expression by competitively binding to matched miRNAs. Thus, negative correlations between lncRNAs and miRNAs or positive correlations between lncRNAs and mRNAs should have been observed. Correlation analysis with the starBase database indicated a positive or negative relationship between each of the four lncRNAs, particularly SNHG17 and HCG18, and COMMD2 or miR-29c-3p (**Figures 6H–O**). By combining expression, survival, and correlation analysis, a CRNDE/LINC00511/SNHG17/HCG18-miR-29c-3p-COMMD2 ceRNA network was constructed (**Figure 6G**) and could potentially serve as a prognostic model in LIHC.

### Relationship Between COMMD2 and Immune Cell Infiltration in LIHC

**Figure 7:**
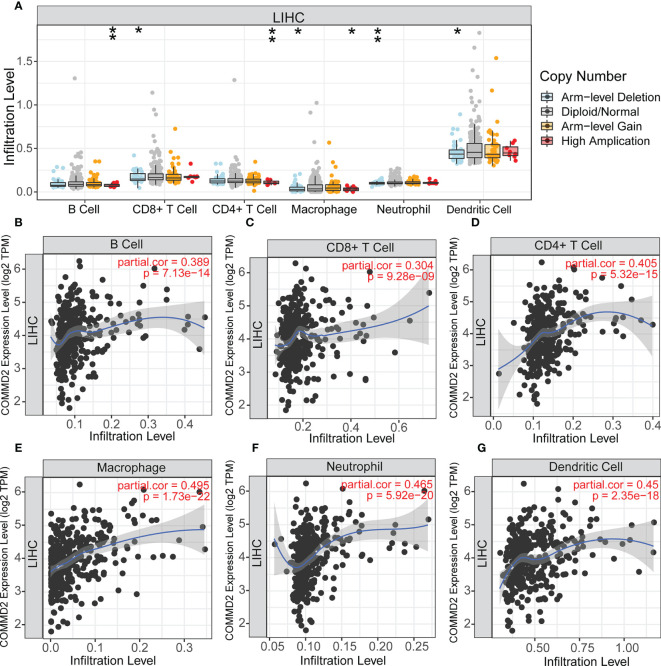
Relationship Between COMMD2 and immune cell infiltration in LIHC. **(A)** Relationship Between COMMD2 copy number alterations and the infiltration level of six dominant immune cells in LIHC. **(B–G)** Correlations of COMMD2 with infiltrating B cells **(B)**, CD8+ T cells **(C)**, CD4+ T cells **(D)**, neutrophils **(E)**, macrophages **(F)** and dendritic cells **(G)** in LIHC. *p < 0.05; **p < 0.01.

**Figure 8:**
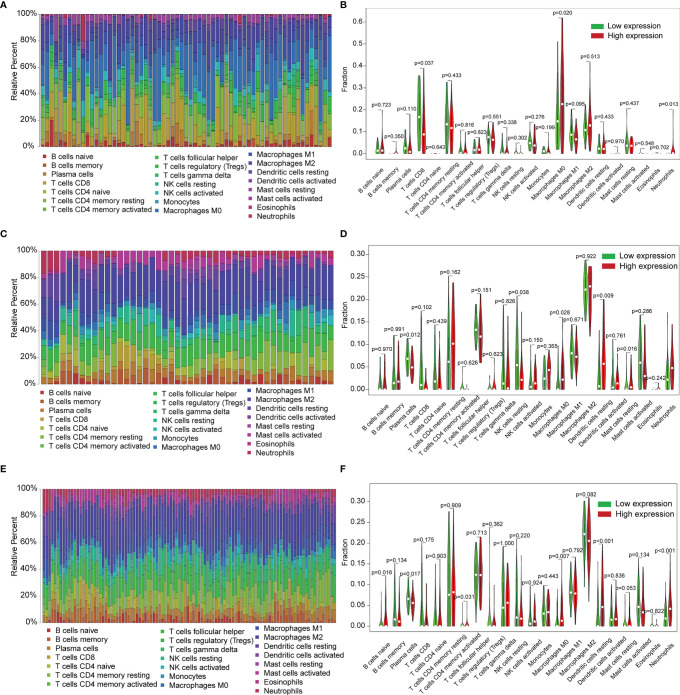
Relationship of COMMD2 expression with 22 immune cell types in LIHC based on CIBERSORT. **(A, C, E)** Relative fractions of 22 immune cell types in the TCGA-LIHC cohort **(A)**, GSE55092 **(C)** and GSE107170 **(E)**. **(B, D, F)** Violin plots of the difference in 22 immune cell types between patients with high and low COMMD2 expression from the TCGA-LIHC cohort **(B)**, GSE55092 **(D)** and GSE107170 **(F)**.

COMMD2 is a member of the COMMD family, the members of which affect the prognosis of patients by participating in the inflammatory response and immune cell infiltration. Therefore, the relationships between COMMD2 and infiltrating immune cells were displayed using TIMER. Positive relationships were observed between the expression of COMMD2 and the infiltration of B cells, CD8+ T cells, CD4+ T cells, macrophages, neutrophils, and dendritic cells (**Figures 7B–G**). Moreover, COMMD2 copy number alterations could affect the infiltration level of six dominant immune cells, especially high amplification and arm-level deletion (**Figure 7A**). To further understand the association between COMMD2 expression and 22 immune cell types in the TCGA-LIHC cohort, we summarized the relative fractions of these immune cells in each LIHC patient using the CIBERSORT method **(** **Figure 8A****)**. Patients with high COMMD2 expression exhibited significantly higher proportions of M0 macrophages and neutrophils (*P <*0.05) and lower proportions of CD8 T cells **(** **Figure 8B****)**. Next, we used two gene expression omnibus (GEO) datasets to determine the above results and found that patients with high COMMD2 expression had a significant increase in the abundance of M0 macrophages, neutrophils, resting dendritic cells and resting mast cells and a significant decrease in the abundance of plasma cells and resting NK cells in GSE55092 **(** **Figures 8C, D****)**. In GSE107170, M0 macrophages, neutrophils, naïve B cells, activated memory CD4 T cells and resting dendritic cells increased and plasma cells decreased in patients with high COMMD2 expression **(** **Figures 8E, F****)**. Thus, high COMMD2 expression is significantly associated with higher proportions of M0 macrophages and neutrophils. All these findings suggested that COMMD2 is closely related to the level of immune infiltration, suggesting that COMMD2 may be involved in regulating LIHC tumor immunity.

### Prognostic Analysis of COMMD2 Expression Based on Immune Cells in LIHC

**Figure 9:**
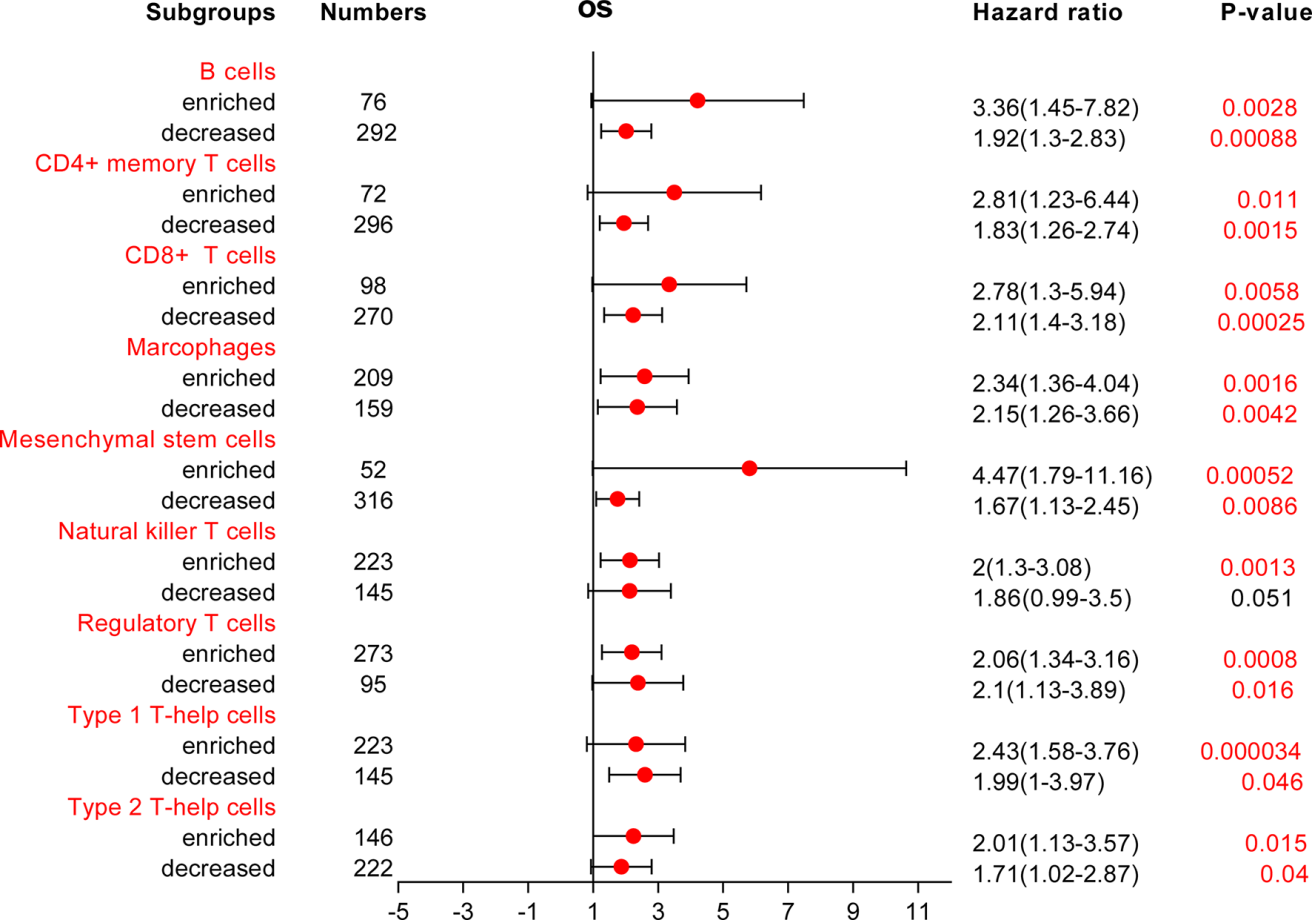
Forest plot of the prognostic value of COMMD2 based on different immune cell subgroups of LIHC patients.

Because COMMD2 expression was significantly associated with immune cell infiltration and a poor prognosis in LIHC, we next investigated whether COMMD2 expression influences the OS of LIHC patients by regulating immune cell infiltration. We performed survival analyses of LIHC patients based on COMMD2 expression in related immune cell subgroups. As shown in **Figure 9** and **Supplementary Figure 4**, different infiltration levels of B cells, CD4+ memory T cells, CD8+ T cells, macrophages, mesenchymal stem cells, regulatory T cells, type 1 T helper cells and type 2 T helper cells were found in LIHC patients, and those with high COMMD2 expression had a poor prognosis. These results indicate that COMMD2 may influence the OS of LIHC patients by regulating immune cell infiltration.

### Correlations of COMMD2 With Immune Cell Biomarkers in LIHC

To further validate the role of COMMD2 in antitumor immunity, we explored the relationships between COMMD2 and immune cell biomarkers in LIHC using the GEPIA database. As listed in **Table 2**, COMMD2 was significantly positively correlated with most immune markers in various immune cell types, including B cells, CD8+ T cells, CD4+ T cells, M1 macrophages, M2 macrophages, neutrophils and dendritic cells. These results further support that COMMD2 is positively related to tumor immune cell infiltration in LIHC.

**Table 2:** Correlation analysis of COMMD2 with immune cell biomarkers in LIHC.

Immune cell	Biomarker	R value	P value
B cells	CD19	0.18	0.00041∗∗∗
	CD79A	0.11	0.034∗
CD8+ T cells	CD8A	0.14	0.0064∗∗
	CD8B	0.062	0.23
CD4+ T cells	CD4	0.31	8.1e−10∗∗∗
M1 macrophages	NOS2	0.22	1.4e−05∗∗∗
	IRF5	0.44	4e−19∗∗∗
	PTGS2	0.27	8e−08∗∗∗
M2 macrophages	CD163	0.031	0.55
	VSIG4	0.17	0.0014∗∗
	MS4A4A	0.18	0.00065∗∗∗
Neutrophils	CEACAM8	0.097	0.063
	ITGAM	0.35	3e−12∗∗∗
	CCR7	0.13	0.01∗∗
Dendritic cells	HLA-DPB1	0.18	0.00044∗∗∗
	HLA-DQB1	−0.035	0.5
	HLA-DRA	0.24	2.4e−06∗∗∗
	HLA-DPA1	0.22	1.6e−05∗∗∗
	CD1C	0.2	7.3e−05∗∗∗
	NRP1	0.46	1.6e−20∗∗∗
	ITGAX	0.34	1.7e−11∗∗∗

∗p value < 0.05, ∗∗p value < 0.01, ∗∗∗p value < 0.001.

### Correlations Between COMMD2 and Immune Checkpoint Molecules in LIHC

**Figure 10:**
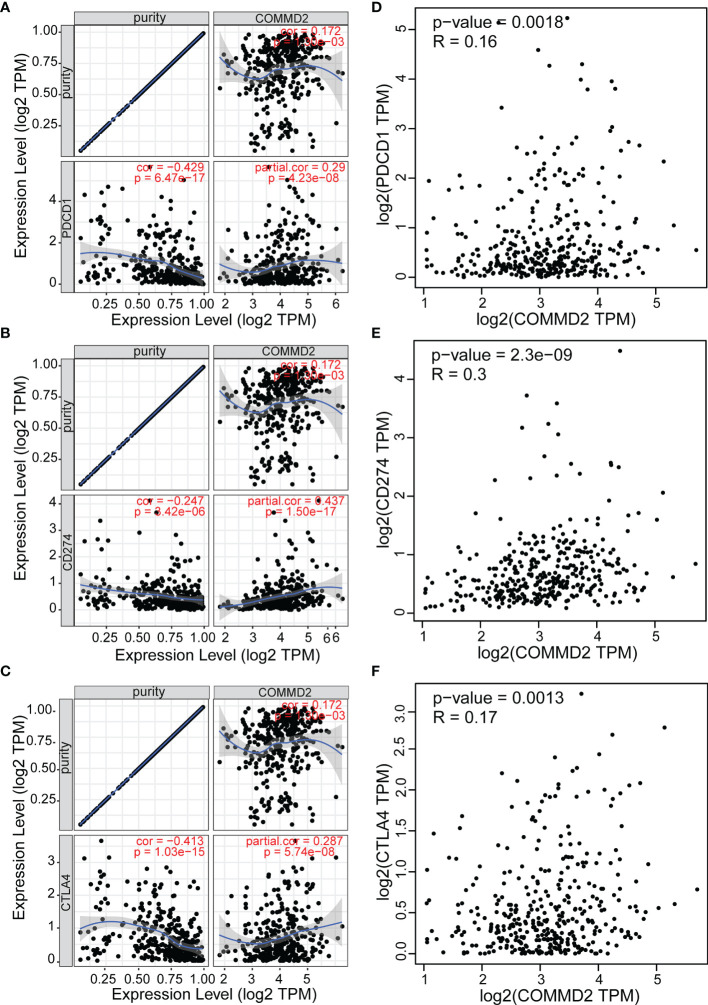
Correlations between COMMD2 and PD-1, PD-L1 and CTLA-4 in LIHC. **(A-C)** Correlations of COMMD2 with PD-1 **(A)**, PD-L1 **(B)** and CTLA-4 **(C)** in LIHC adjusted for purity using TIMER. **(D-F)** Relationships of COMMD2 with PD-1 **(D)**, PD-L1 **(E)** and CTLA-4 **(F)** in LIHC as determined using the GEPIA database.

As crucial immune checkpoint molecules, programmed death-1 (PD1), programmed death-ligand 1 (PD-L1) and cytotoxic T ymphocyte antigen-4 (CTLA-4) play important roles in tumor immune escape. Based on the potential oncogenic role of COMMD2 in LIHC, the correlations of COMMD2 with PD1, PD-L1 and CTLA-4 were estimated. COMMD2 was significantly positively correlated with PD1, PD-L1 and CTLA-4 in LIHC (**Figures 10A–C**). Similar results were found using the GEPIA database, which revealed significant positive correlations of COMMD2 with PD1, PD-L1 or CTLA 4 in LIHC **(** **Figures 10D–F**). These results demonstrate that COMMD2 may be involved in tumor immune escape during LIHC tumorigenesis.

### Effects of COMMD2 Knockdown on the Proliferation and Migration of LIHC Cells *In Vitro*

**Figure 11:**
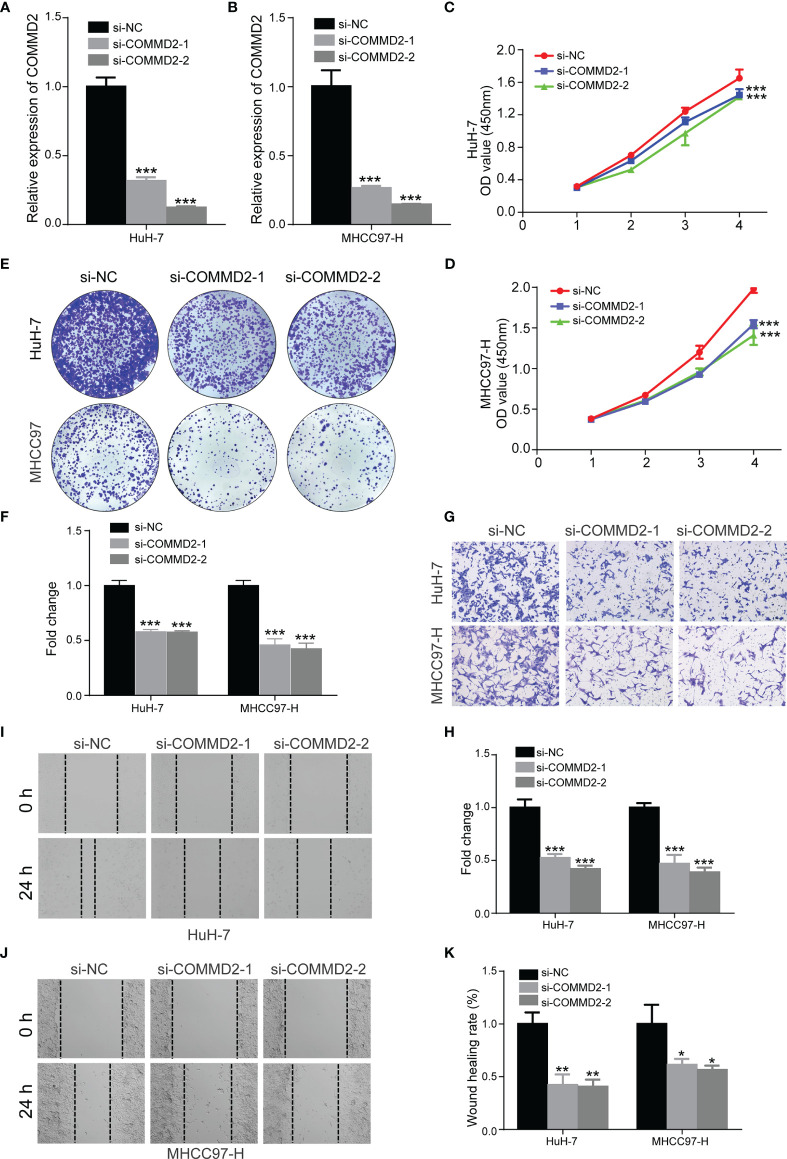
Effects of COMMD2 knockdown on the proliferation and migration of LIHC cells. **(A, B)** The efficiency of COMMD2 downregulation in HuH-7 **(A)** and MHCC97-H **(B)** cells after COMMD2 siRNA transfection was detected by RT-qRCR. **(C–F)** Effect of COMMD2 knockdown on the proliferation of HuH-7 and MHCC97-H cells as determined by CCK8 **(C, D)** and colony formation assays **(E, F)**. **(G–K)** Effect of COMMD2 knockdown on the migration of HuH-7 and MHCC97-H cells as detected by transwell **(G, H)** and wound healing assays **(I–K)**. Scale bar=50 μm. *P < 0.05; **P < 0.01; ***P < 0.001.

To assess the function of COMMD2 in LIHC, we knocked down its expression in HuH-7 and MHCC97-H cells using siRNAs, and the silencing efficiency was determined by RT-qPCR (**Figures 11A, B**). CCK8 and colony formation assays were performed to explore the effect of COMMD2 knockdown on LIHC cell proliferation, revealing that the proliferation of HuH-7 and MHCC97-H cells was significantly decreased after COMMD2 downregulation (**Figures 11C–F**). Subsequently, to investigate the impacts of COMMD2 knockdown on LIHC cell migration ability, wound healing and transwell assays were performed, demonstrating that COMMD2 knockdown drastically decreased the migration ability of HuH-7 and MHCC97-H cells compared with that of control group cells (**Figures 11G–K**).

## Discussion

LIHC is the third leading cause of cancer-related mortality worldwide (1). Although various therapeutic strategies have been adopted for LIHC patients, their efficacies remain unsatisfactory (23). Identifying novel biomarkers of malignant LIHC is essential to identify new effective therapeutic targets and improve LIHC patient prognosis. Increasing evidence has demonstrated that COMMD proteins play key roles in the development and progression of multiple human cancers (12, 13), including LIHC. However, the underlying mechanisms and clinical value of COMMD2 and its correlation with immune cell infiltration in LIHC remain unknown.

In the present study, we first performed pancancer analysis of COMMD2 expression using the TIMER and UALCAN databases, and found that COMMD2 was abnormally expressed in the tumor tissues of 13 different cancer types compared with that in the corresponding normal tissues. Association analyses of COMMD2 with the survival of patients with candidate types of cancer indicated that high COMMD2 expression was associated with a poor prognosis in LIHC. Clinical association analyses demonstrated that increased COMMD2 expression was correlated with higher histological grade, more advanced clinical stage, lymph node metastasis and the TP53 mutation status in LIHC patients. Furthermore, COMMD2 knockdown suppresses LIHC cell proliferation and migration *in vitro via* a series of functional assays.

Previous studies have revealed that ncRNAs, particularly miRNAs, lncRNAs, and circular RNAs, are involved in the development and progression of tumors through gene regulation mechanisms involving ceRNA regulatory networks (24–27). To explore the upstream miRNAs that modulate COMMD2 expression, we used four prediction programs to predict miRNAs that potentially bind COMMD2 and ultimately identified 21 miRNAs. Most of these miRNAs play suppressor roles in LIHC. For example, miR−29b-3p regulates the TGF−β1 and p53 signaling pathways to inhibit the growth and induce the apoptosis of LIHC ascites H22 cells (28). miR-29a-3p inhibits cell proliferation and migration by targeting PTEN and thereby regulating the NF-kappaB pathway in LIHC (29). miR-29c-3p inhibits tumor progression by regulating the methylation of DNMT3B and LATS1 in LIHC (30). Among the 21 identified candidate miRNAs, only miR-29c-3p was expressed at a low level, which was negatively correlated with the high expression of COMMD2 and associated with a better prognosis for LIHC patients as determined by the combination of expression, correlation, and survival analyses. Thus, miR-29c-3p was selected as the most promising upstream miRNA of COMMD2. Previous studies also showed that miR-29c-3p inhibits LIHC proliferation (31).

According to the ceRNA hypothesis (32), the potential lncRNAs upstream of the miR-29c-3p/COMMD2 axis should be oncogenic lncRNAs in LIHC. Subsequently, lncRNAs upstream of the miR-29c-3p/COMMD2 axis were also predicted, ultimately identifying 54 possible lncRNAs. By performing expression, correlation, and survival analyses, CRNDE, LINC00511, SNHG17 and HCG18 were identified as the most promising upregulated lncRNAs. High expression of CRNDE, LINC00511, SNHG17 and HCG18, which have positive and negative relationships with COMMD2 and miR-29c-3p, respectively, was associated with a poor prognosis in LIHC. The four lncRNAs functioned as oncogenes in multiple tumors, including LIHC. For example, the lncRNA CRNDE facilitates the proliferation, invasion, migration and chemoresistance of LIHC (33–36). LINC00511 promotes malignant cell behaviors and correlates with prognosis in LIHC (37–39). The lncRNA SNHG17 promotes cell proliferation and migration and predicts a poor prognosis in LIHC (40, 41). The lncRNA HCG18 contributes to the progression of LIHC (42, 43). Thus, the CRNDE/LINC00511/SNHG17/HCG18-miR-29c-3p-COMMD2 axis regulates the development and progression of LIHC.

Numerous recent studies have confirmed that tumor immune cell infiltration influences tumor angiogenesis and the prognosis of patients with LIHC (44–48). Herein, we found that COMMD2 was significantly positively correlated with various infiltrating immune cells in LIHC, especially M0 macrophages and neutrophils by various database. Moreover, COMMD2 was closely related to immune markers of these tumor-infiltrating immune cells. Importantly, COMMD2 was shown to influence the OS of LIHC patients through immune cell infiltration. These findings indicate that tumor immune infiltration may partially explain the carcinogenic effect of COMMD2 in LIHC. In addition, immune checkpoint molecules, including PD-1, PD-L1, and CTLA-4, are associated with the prognosis of LIHC patients (49–51). Checkpoint inhibitors (CPIs) targeting PD-1, PD-L1, or CTLA-4 have led to clinical breakthroughs in oncological treatment (52–55). Thus, we also assessed the relationships between COMMD2 and immune checkpoint molecules. High COMMD2 expression was significantly linked to PD1, PD-L1 and CTLA-4 levels in LIHC, suggesting that targeting COMMD2 might enhance immunotherapeutic efficacy in LIHC.

However, some limitations in our study should be considered. First, our finding is mainly relies on public databases, more data and larger LIHC cohorts were required to validate its clinical suitability. Second, the role of COMMD2 in tumor immune infiltration needs to be further confirmed *in vitro* or *in vivo*. Finally, the carcinogenic mechanism of the CRNDE/LINC00511/SNHG17/HCG18-miR-29c-3p-COMMD2 axis in LIHC requires more functional studies to elucidate. Therefore, further investigations, including basic experiments and clinical trials, are needed to perform in the future.

**Figure 12:**
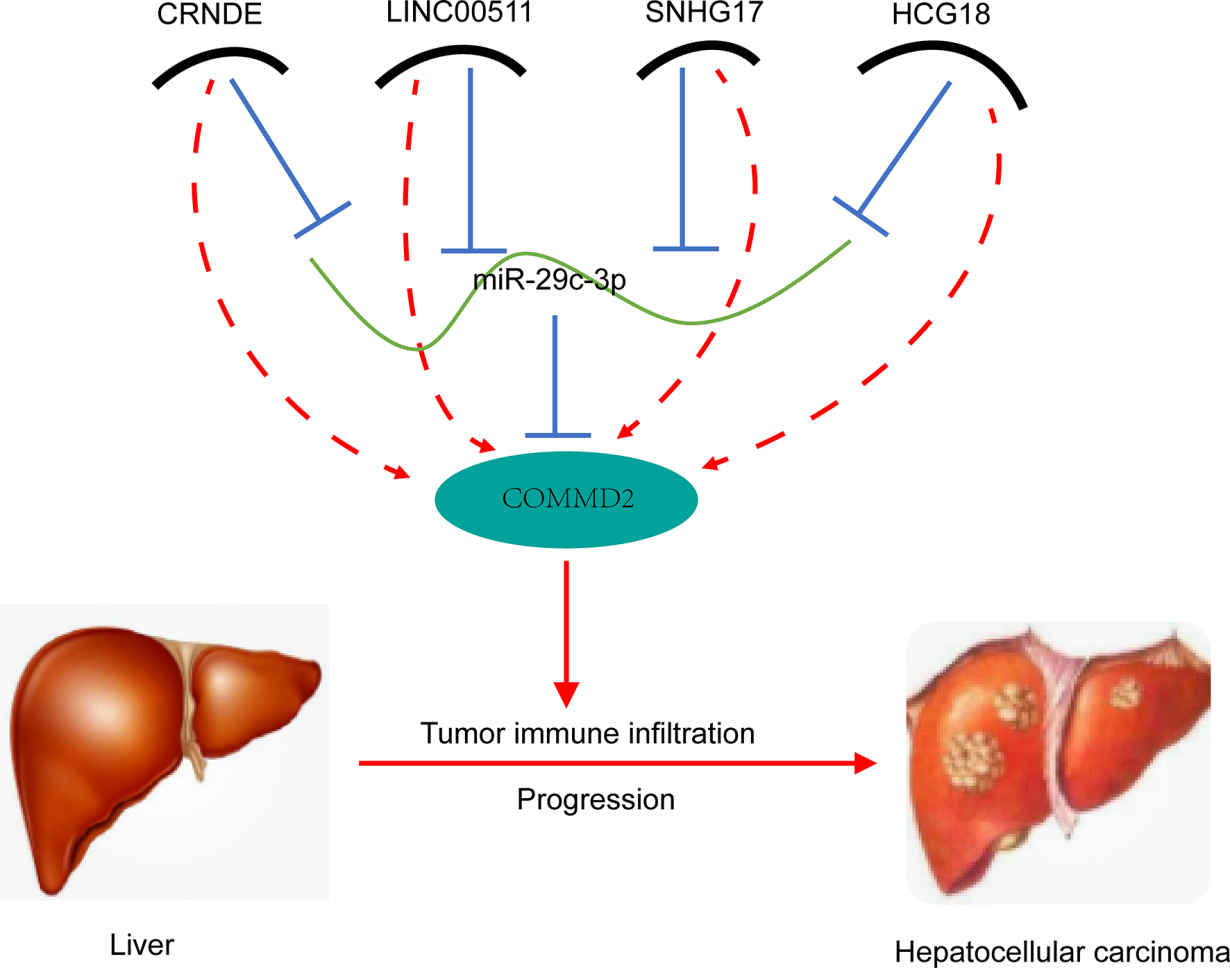
Model of the CRNDE/LINC00511/SNHG17/HCG18-miR-29c-3p-COMMD2 axis in LIHC carcinogenesis.

In conclusion, our results indicate a carcinogenic effect of COMMD2 and its potential as a novel prognostic biomarker in LIHC. Furthermore, we further elucidated the underlying oncogenic mechanism of COMMD2 by constructing a CRNDE/LINC00511/SNHG17/HCG18-miR-29c-3p ceRNA network in LIHC (**Figure 12**). Additionally, our study showed that COMMD2 might play a cancer-promoting role by regulating tumor immune cell infiltration in patients with LIHC. Therefore, these findings provide a potentially valuable target for LIHC prognosis and immunotherapy.

## Publisher’s Note

All the claims expressed in this article are solely those of the authors and do not necessarily represent those of their affiliated organizations, or those of the publisher, editors and reviewers. Any product that may be evaluated in this article, or claim that may be made by its manufacturer, is not guaranteed or endorsed by the publisher.

## Data Availability Statement

The original contributions presented in the study are included in the article/**Supplementary Material**. Further inquiries can be directed to the corresponding authors.

## Ethics Statement

This study was approved by the Ethics Committee of the First Affiliated Hospital of Nanchang University. The patients/participants provided their written informed consent to participate in this study.

## Author Contributions

LZ and JX designed this work. WF conducted the experiments and collected the data. WF and YG analyzed the data. WF and LZ drafted the manuscript. JX revised the manuscript. All the authors approved the final version of this manuscript.

## Funding

This work was supported by the National Natural Science Foundation of China (grant number 81760431 and 81860427) and the Natural Science Foundation of Jiangxi Province, China (grant number 20161BAB205243).

## Conflict of Interest

The authors declare that the research was conducted in the absence of any commercial or financial relationships that could be construed as a potential conflict of interest.

## Publisher’s Note

All claims expressed in this article are solely those of the authors and do not necessarily represent those of their affiliated organizations, or those of the publisher, the editors and the reviewers. Any product that may be evaluated in this article, or claim that may be made by its manufacturer, is not guaranteed or endorsed by the publisher.
