# Supplementary figures and images for: COMMD2 Upregulation Mediated by an ncRNA Axis Correlates With an Unfavorable Prognosis and Tumor Immune Infiltration in Liver Hepatocellular Carcinoma
Source: Front Oncol. 2022 Apr 29;12:853026. doi: 10.3389/fonc.2022.853026 (PMC9099436; doi:10.3389/fonc.2022.853026)

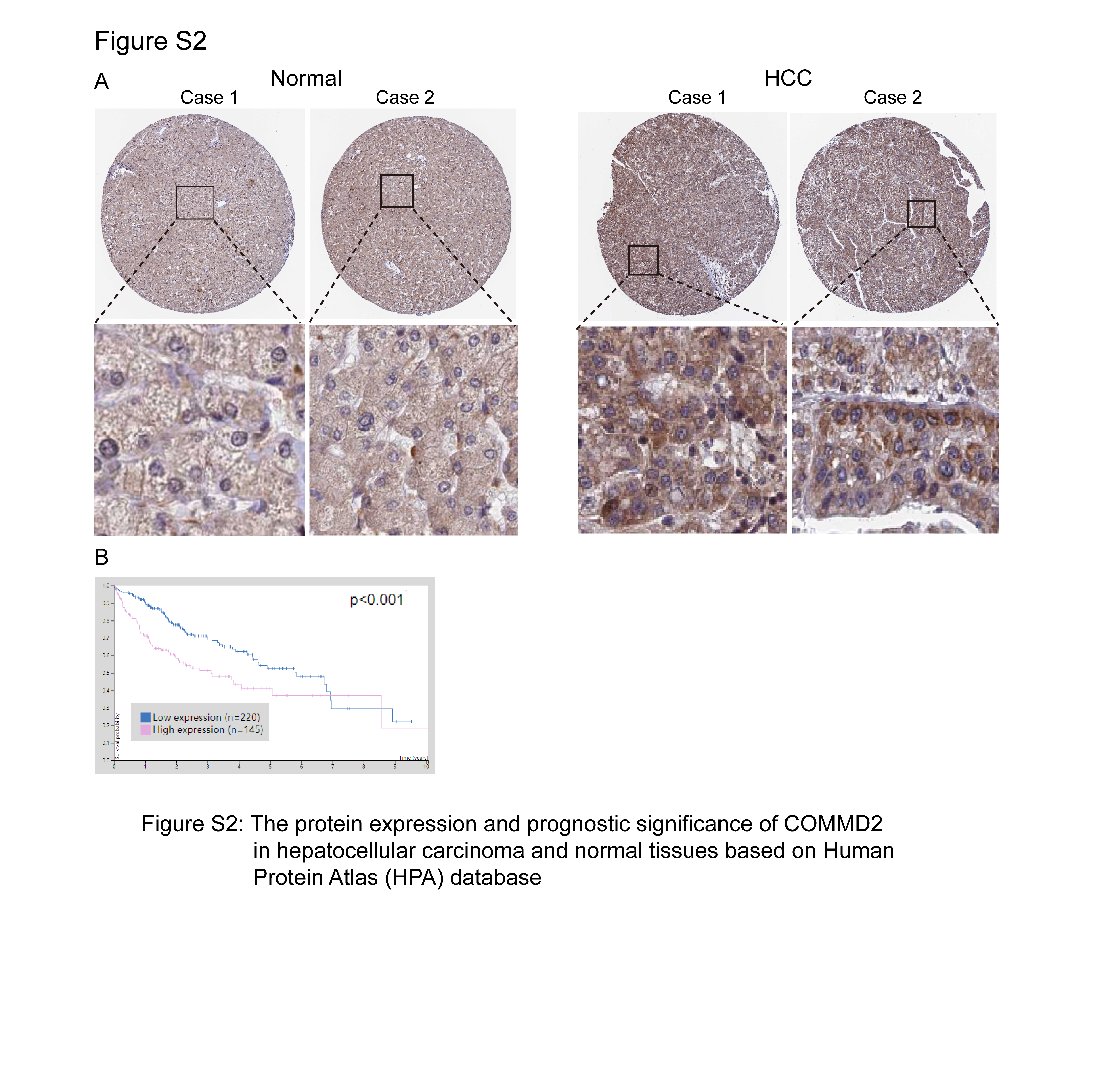

Supplement: Supplementary file 1 [file DataSheet_1.zip › figure S2.tif]

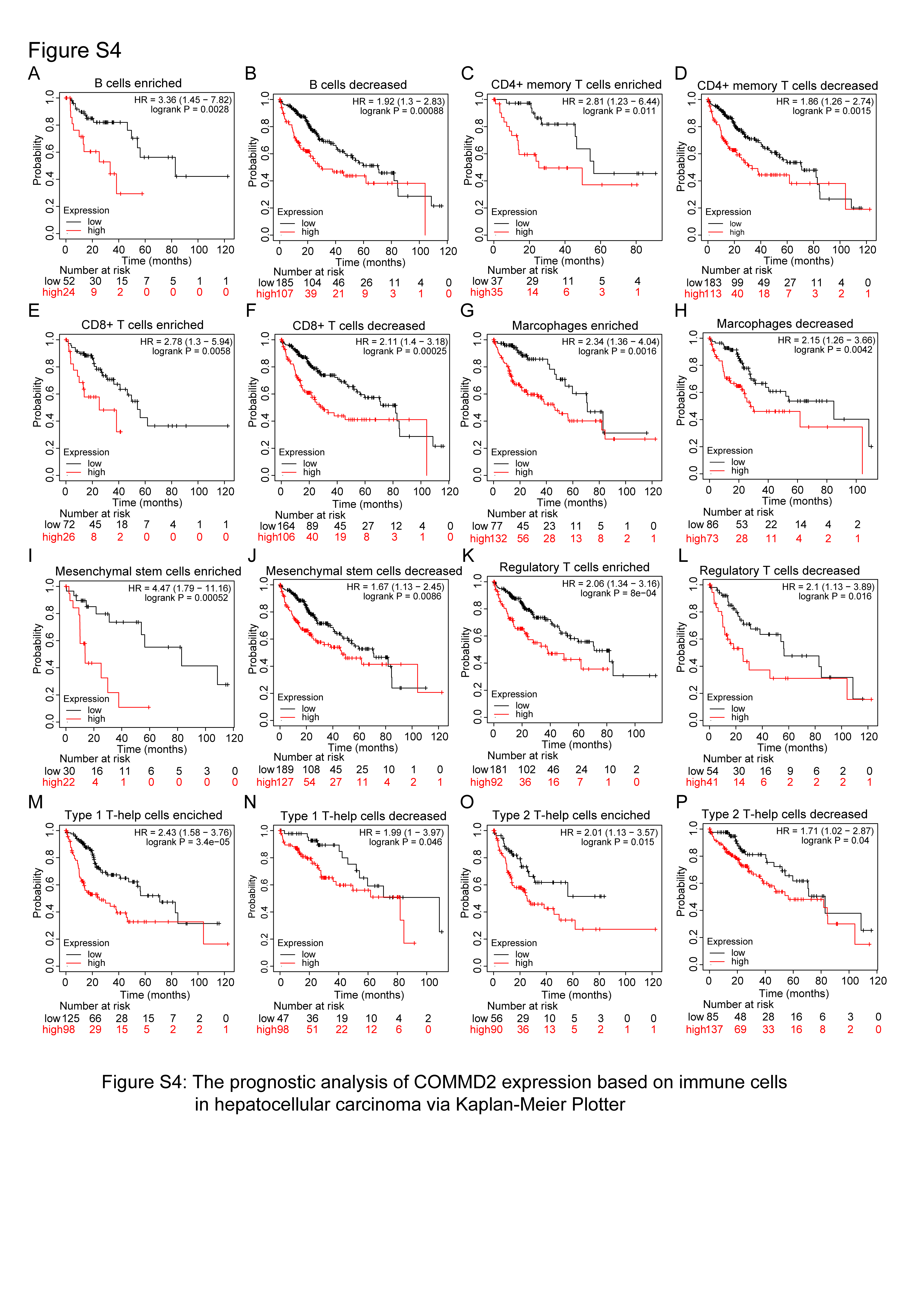

Supplement: Supplementary file 1 [file DataSheet_1.zip › figure S4.tif]

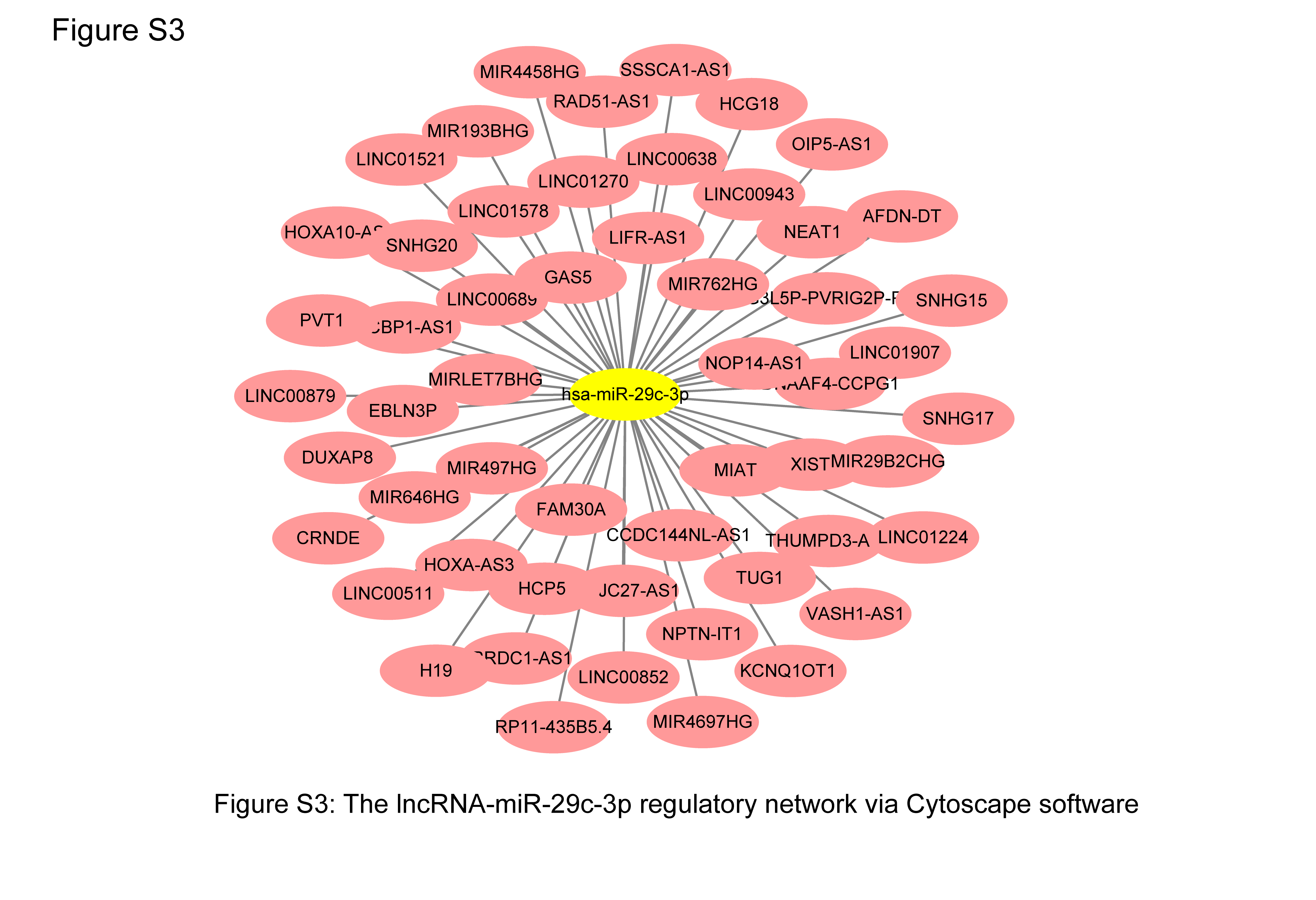

Supplement: Supplementary file 1 [file DataSheet_1.zip › figureS3.tif]

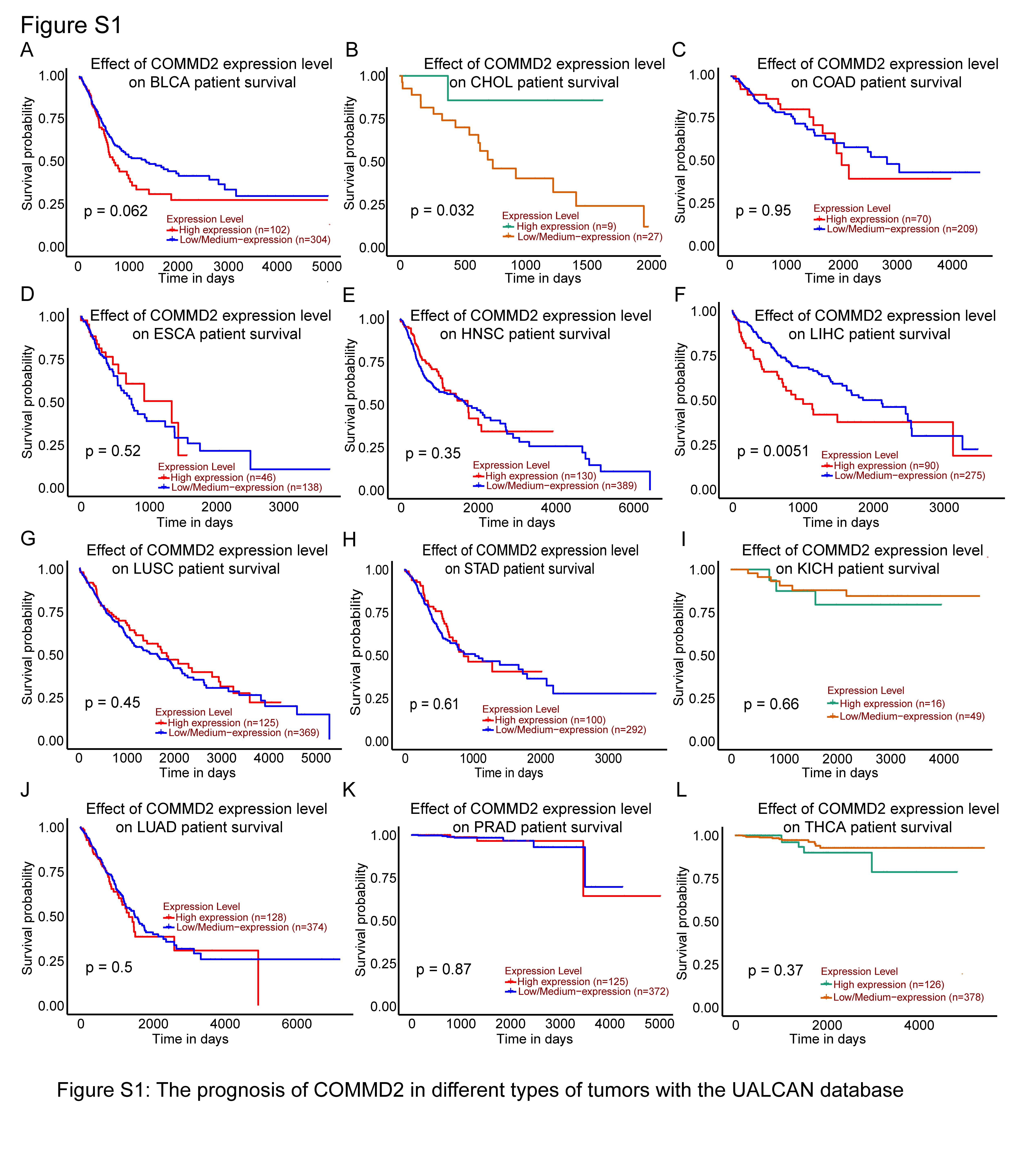

Supplement: Supplementary file 1 [file DataSheet_1.zip › figure S1.tif]

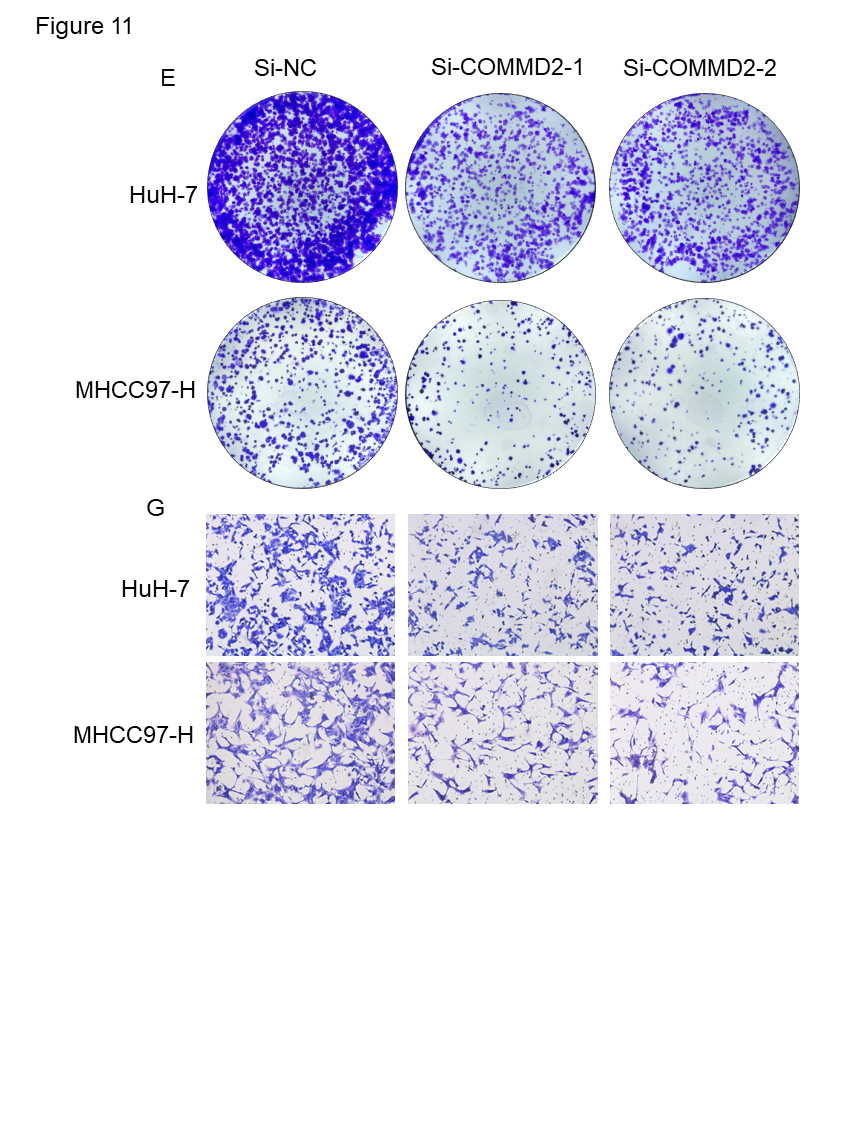

Supplement: Supplementary file 2 [file DataSheet_2.zip › additional file/figrue 11.tif]

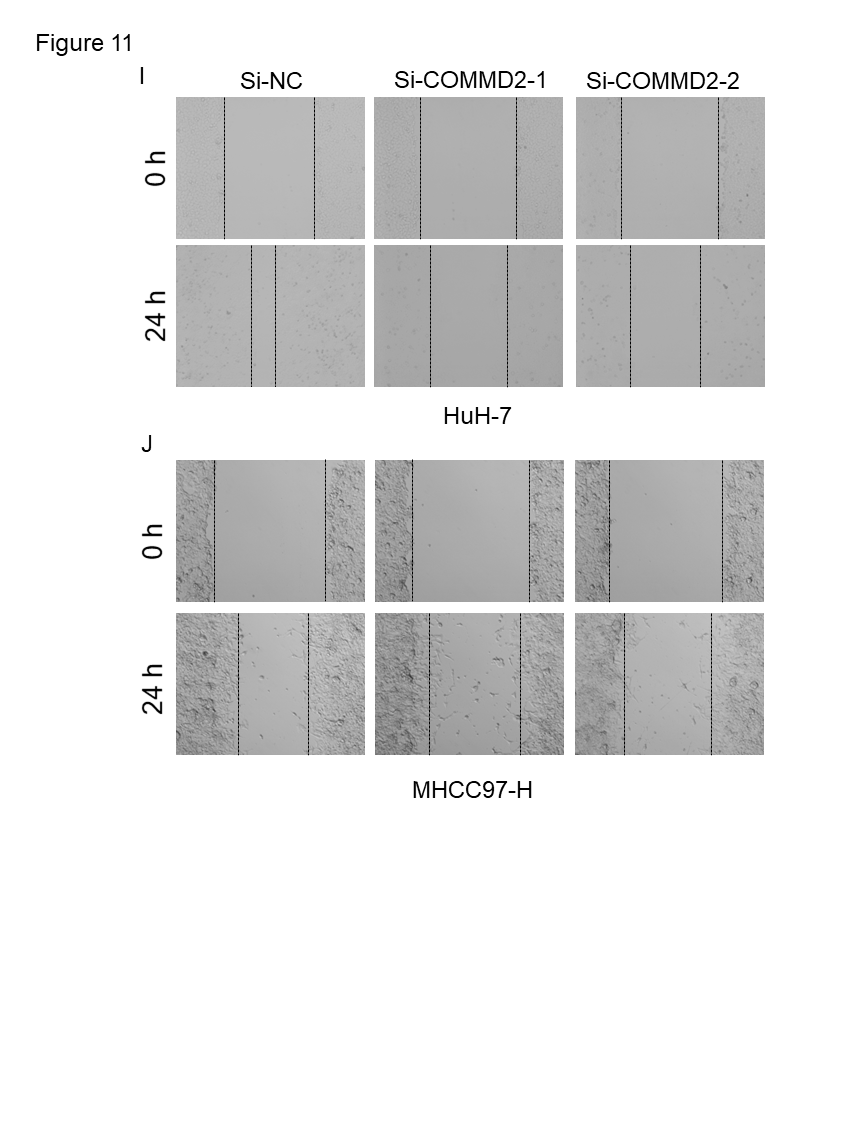

Supplement: Supplementary file 2 [file DataSheet_2.zip › additional file/figure 11-2.tif]
