# Supplementary material for: COMMD2 Upregulation Mediated by an ncRNA Axis Correlates With an Unfavorable Prognosis and Tumor Immune Infiltration in Liver Hepatocellular Carcinoma
Source: Front Oncol. 2022 Apr 29;12:853026. doi: 10.3389/fonc.2022.853026 (PMC9099436; doi:10.3389/fonc.2022.853026)
Supplement: Supplementary file 2 [file DataSheet_2.zip › additional file/Jianguoyun.docx]

GSE55092：<https://www.jianguoyun.com/p/DRpC3IEQnsavChiArrME>

GSE107170：<https://www.jianguoyun.com/p/DdJCLroQnsavChiMrrME>

TCGA-LIHC：<https://www.jianguoyun.com/p/DbYxzrkQnsavChiWrrME>
